# A case of retrograde colonic intussusception by tubulovillous adenoma

**DOI:** 10.1007/s12328-025-02205-z

**Published:** 2025-08-25

**Authors:** Michiko Iki, Nobuhiko Kanaya, Ryohei Shoji, Yoshihiko Kakiuchi, Yoshitaka Kondo, Shinji Kuroda, Kunitoshi Shigeyasu, Shunsuke Kagawa, Toshiyoshi Fujiwara

**Affiliations:** Department of Gastroenterological Surgery, Okayama University Graduate School of Medicine, Dentistry and Pharmaceutical Sciences, 2-5-1 Shikata-cho, Kita-ku, Okayama, 700-8558 Japan

**Keywords:** Retrograde colonic intussusception, Colonic polyp, Multiple system atrophy, Shy–Drager syndrome

## Abstract

**Introduction:**

Retrograde colonic intussusception is a rare condition in adults, often caused by organic lesions such as tumors. Autonomic dysfunction in disorders like multiple system atrophy (MSA) might contribute to its occurrence.

**Case presentation:**

An 81-year-old bedridden woman with a history of MSA presented with severe abdominal pain and abdominal distension lasting 4 days. She had chronic severe constipation managed with laxatives and manual disimpaction. CT imaging revealed retrograde intussusception of the rectum into the sigmoid colon. Endoscopic reduction was attempted but was unsuccessful due to scope impassability. Emergency laparotomy identified a 4–5 cm tumor at the lead point, and manual reduction resulted in bowel perforation. Hartmann’s procedure with D2 lymphadenectomy was performed. The tumor was histopathologically diagnosed as a tubulovillous adenoma with no malignant features. The patient’s postoperative recovery was uneventful except for a urinary tract infection (Clavien–Dindo Grade II), and she was transferred to a rehabilitation facility on postoperative day 24.

**Conclusion:**

Failure of reduction by air enema should raise suspicion for retrograde intussusception, warranting prompt surgery if an organic lead point is suspected.

## Introduction

Intussusception is a condition where a part of the intestine folds into another section of the intestine, leading to obstruction of the bowel, reduced blood supply to the affected portion, and tissue damage if not treated promptly [1]. It is most commonly seen in children, particularly those under 2 years of age, but can also occur in adults, where it is often secondary to underlying conditions such as tumors, or postoperative adhesions [2]. Adult intussusception mainly occurs in the small intestine but sometimes colon with a higher percentage of malignancy in the colon [3]. Unlikely children, surgical intervention in adults is required if enema reduction fail.

Retrograde intussusception, where the distal bowel telescopes into the proximal segment, is an exceptionally rare condition in adult intussusception cases [4]. It is often secondary to an organic lesion such as a polyp or tumor. Multiple system atrophy (MSA) is a neurodegenerative disorder characterized by autonomic dysfunction, including chronic constipation, which may predispose patients to this condition [5]. We report a case of retrograde colonic intussusception by tubulovillous adenoma under the chronic constipation in a patient with MSA and review relevant literatures.

## Case presentation

**Fig. 1:**
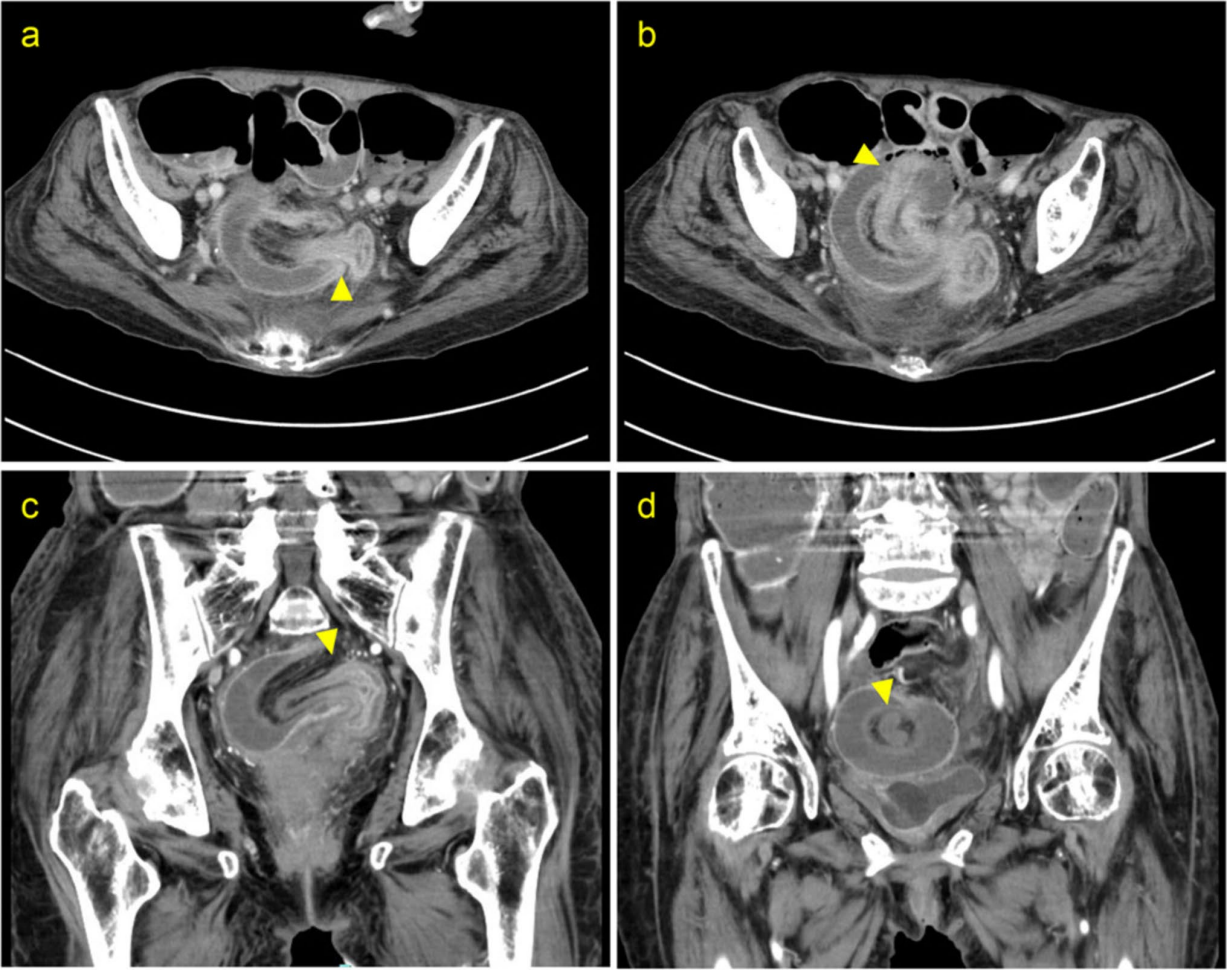
Images of enhanced computed tomography (CT) showing retrograde colonic intussusception. **a, b** Axial view demonstrating the intussusception. **c, d** Coronal view highlighting the telescoped bowel segment. The intussuscepted segment is marked with a yellow triangle.

**Fig. 2:**
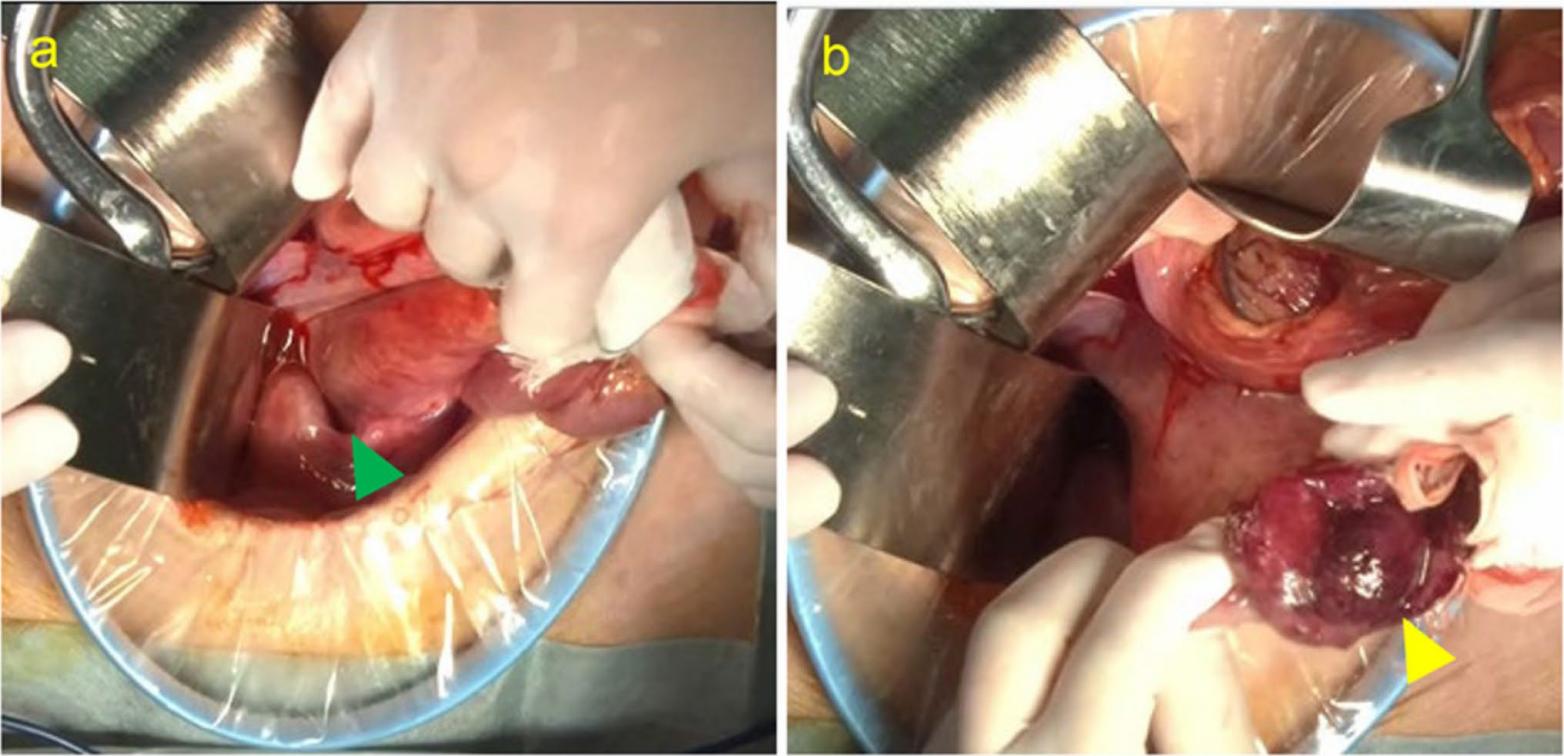
Intraoperative findings of retrograde colonic intussusception. **a** The intussuscepted colonic segment (green triangle) is identified and gently exteriorized through the surgical field. **b** The resected colonic segment with the tumor, showing ischemic changes. The tumor is marked with a yellow triangle.

**Fig. 3:**
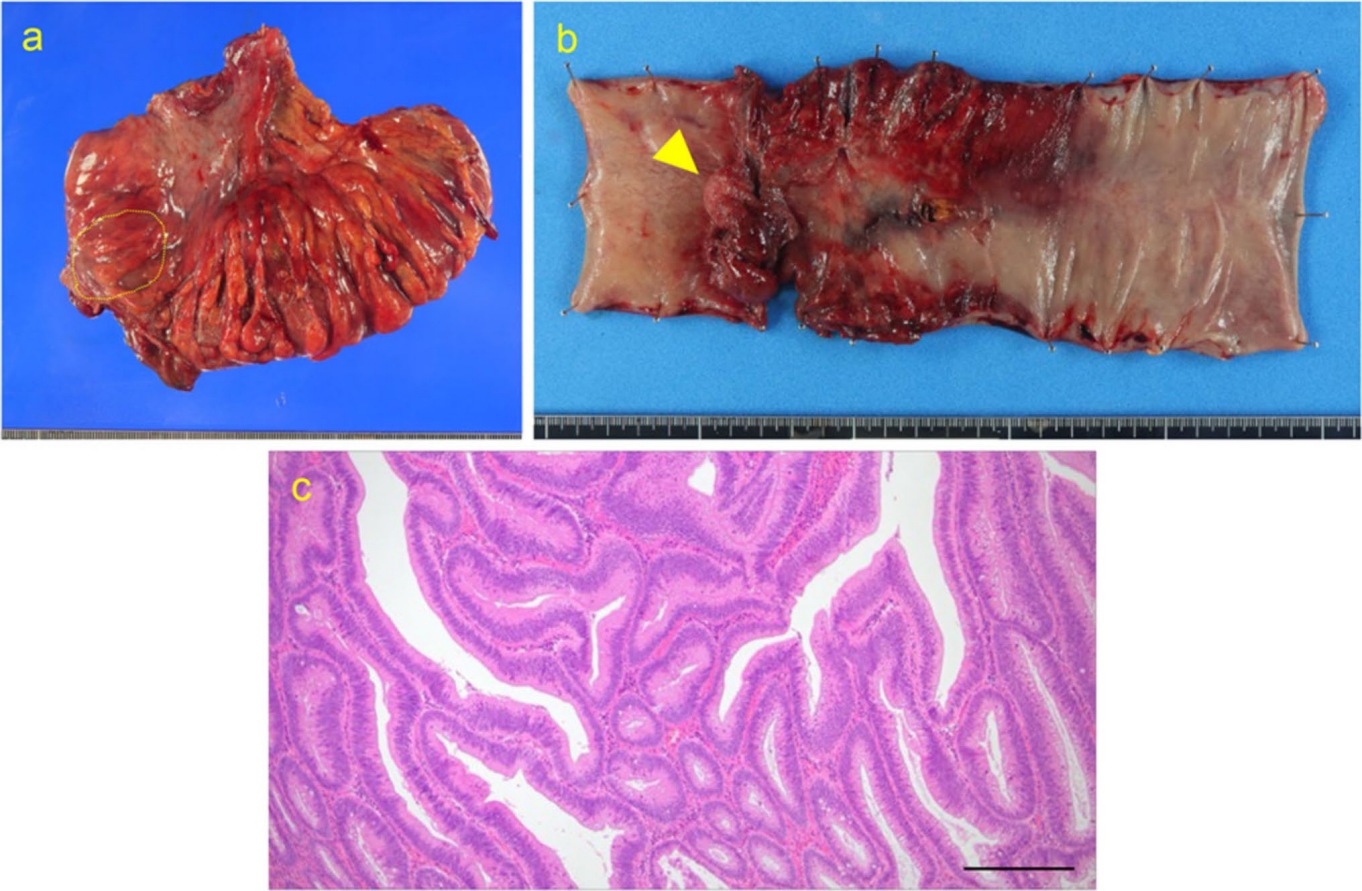
Resected specimen of sigmoid colon. **a** Gross appearance of resected specimens. The tumor is marked around a yellow dotted line. **b** Gross appearance of the resected colon and tumor. The tumor is marked with a yellow dotted line. Necrotic changes are evident in the proximal colon to the tumor. **c** Histopathological examination using hematoxylin and eosin staining, confirming tubulovillous adenoma.

An 81-year-old woman with a history of MSA presented with abdominal pain and distension of 4 days’ duration. Her medical history included severe constipation managed with laxatives and manual disimpaction. She was bedridden and dependent on home oxygen therapy. Then abdominal computed tomography (CT) revealed retrograde intussusception from the sigmoid colon to the rectosigmoid junction, with dilation of the proximal bowel. (Fig. 1). Colonoscopy was performed to assess the localization of tumors at the site of retrograde intussusception, but no tumor was observed. Due to the obstruction, the intussuscepted segment could not be visualized, and endoscopic reduction was unsuccessful. Then emergency laparotomy underwent at the same day. Surgical exploration revealed rectosigmoid intussusception with a 4–5 cm tumor at the lead point (Fig. 2). Manual reduction was attempted but resulted in perforation. Given the oncologic and surgical considerations, Hartmann’s procedure with D2 lymphadenectomy was performed. The patient postoperatively developed a urinary tract infection (Clavien–Dindo Grade II) but otherwise recovered well and was discharged to a rehabilitation facility on postoperative day 24. Histopathological examination confirmed a tubulovillous adenoma without lymph node metastasis and the intussuscepted colon showing necrosis (Fig. 3).

## Discussion

We presented a rare case of retrograde colonic intussusception by tubulovillous adenoma in an 81-year-old woman with a history of MSA. The condition was caused by a tubulovillous adenoma in the sigmoid colon, compounded by the patient’s severe chronic constipation and autonomic dysfunction. Emergency surgery was required after failed endoscopic reduction.

Retrograde intussusception, characterized by distal bowel telescoping into proximal segments, is particularly rare (0.25–1%) [4, 6]. In children, the condition is often idiopathic, caused by immature bowel fixation or lymphoid hyperplasia secondary to viral infections [4]. In adults, however, the majority of cases have an identifiable organic cause, such as tumors, postoperative changes, or other structural abnormalities. A literature search for retrograde intussusception between 2010 and 2025 using the keywords “retrograde”.

and “intussusception” in PubMed revealed 65 cases of retrograde intussusception, including our case. The review of 65 cases of retrograde intussusception in the literature identified post-stomach-associated surgeries such as Roux-en-Y gastric bypass (43 cases), tumors (6 cases), tubes such as feeding tubes (3 cases), pancreaticoduodenectomy (3 cases), myotomy (3 cases) and other reasons (7 cases) [7–26]. Therefore, the majority of cause was by gastrectomy-associated reasons. In our case, the intussusception was directly caused by the tumor at the lead point, consistent with these findings. Of the 6 cases who had retrograde intussusception induced by tumors, 3 cases had tumors at the colon (Table 1) [12, 14, 21, 27–29]. Among the 6 cases, none had a documented medical history of neurological disease, although one case presented with constipation. Surgery was performed in 6 cases. In these cases, preoperative diagnosis was confirmed via CT scan, and non-operative reduction was unsuccessful. Similarly, in our case, preoperative CT findings showed characteristic features of retrograde intussusception, including an inverted target sign and proximal bowel dilation, allowing for an accurate diagnosis before surgery. Endoscopic reduction was unsuccessful, and surgery was selected as the definitive treatment.

**Table 1:** Summary of the six cases of retrograde intussusception caused by tumor

Year	Author	Age	Sex	Chief complaints	Treatments	Location	Medical history	Etiology
2013	Shibahara et al.	36	F	Abdominal pain and vomiting	Endoscopic polypectomy and hand-assisted laparoscopic reduction	Sigmoid into descending colon		Tubular adenoma
2014	Destro et al.	5	M	Abdominal pain and constipation	Surgery (manual reduction and resection of small bowel)	Ileal tract	None	Sub-mucous intestinal lipoma
2017	Egbuchulem et al.	0 (11 month)	M	Passage of watery stools, postprandial vomiting, and progressive abdominal distension	Surgery (resection of devitalized bowel)	Sigmoid colon into descending colon		Reactive follicular hyperplasia
2019	Aldossary et al.	45	F	Nothing (detected incidentally)	Surgery (pancreas-sparing duodenectomy)	Duodenum	Chronic anemia	Brunner’s gland hamartoma
2022	Vutukuru et al.	11	F	Abdominal pain, vomiting and abdominal distension	Surgery (jejunum resection)	Jejuno-duodenal intussusception	Peutz–Jeghers syndrome	Peutz–Jeghers syndrome polyps
2023	Fukuda et al.	78	F	Abdominal pain and melena	Surgery (laparoscopic transverse colon resection with lymph node dissection)	Transverse colon near the splenic flexure		Adenocarcinoma

*M* male, *F* female

In cases with cystic fibrosis, chronic constipation might lead to the formation of fecaliths. These fecaliths can serve as a lead point for intussusception, as described by Adewale et al. [30]. In our case, chronic constipation might play a crucial role in the development of retrograde intussusception because chronic constipation increases intraluminal pressure, and the presence of a sigmoid colon tumor likely obstructed normal peristalsis, creating conditions favorable for retrograde intussusception. This hypothesis is supported by the CT findings at admission, which showed moderate bowel distension and fecal stasis. Furthermore, the patient’s history of MSA might contribute to the pathogenesis. MSA is a neurodegenerative disorder that involves autonomic dysfunction, including gastrointestinal dysmotility [5]. Studies have shown delayed gastric emptying and prolonged colonic transit times in patients with MSA compared to healthy individuals [5]. In our case, autonomic dysfunction likely impaired normal peristalsis, creating a predisposition to chronic constipation and abnormal bowel dynamics. In addition, the patient’s severe orthostatic hypotension and bedridden status further exacerbated gastrointestinal dysmotility. Beyond MSA, other conditions that involve autonomic dysfunction, such as menopausal syndrome or depression, may also increase the risk of retrograde intussusception [5, 31]. Elevated intraluminal pressure, often resulting from chronic constipation, maintaining effective bowel management might be a key strategy in preventing this rare condition.

## Conclusion

Retrograde intussusception is difficult to diagnose preoperatively due to its rarity and atypical presentation. In cases where the intussuscepted mass does not reduce with air enema or remains fixed despite increasing pressure, the possibility of retrograde intussusception should be considered. Prompt surgical intervention is essential, especially when an organic lesion is suspected as the lead point.

## Abbreviations

MSA: Multiple system atrophy
CT: Computed tomography
